# Acute Rheumatism in a Child

**Published:** 1880-04

**Authors:** Barton Dozier

**Affiliations:** Ukiah City, Cal.


ARTICLE IV.

### ACUTE RHEUMATISM IN A CHILD.

BY BARTON DOZIER, M. D., OF UKIAH CITY, CAL.

I beg to report the following case of acute rheumatism, solely on account of its extreme rarity in so young a child.

Was called March 21st about three miles in the country to see infant child (boy) of Mr. F., aged nine months. On my arrival found the child with considerable fever and very fretful; would cry out as though suffering excruciating pain. The mother gave the history of the case, which may be briefly stated as follows, viz:

"Two nights ago the baby woke me up, crying as if something had hurt it; tried to soothe it to sleep, but my efforts seemed only to increase its anguish; it vomited several times, and vomits now whenever it takes its milk; the left knee is red, swollen and hot."

On examination I found the left leg considerably swollen and flushed, and the knee and hip joints of the extremity acutely sensitive to the least pressure or movement. After satisfying myself as to the correct diagnosis of the case, I prescribed Dover's powder in small and repeated doses, and an anodyne liniment to be applied to the affected joints; the limb kept closely wrapped in flannel, also lime water to be given in the milk, as all food taken upon the stomach had been vomited for the past twelve hours. Visited the little patient next day and found that he had rested better the previous night; leg and joints still swollen and red and tender to the touch, but the stomach quite relieved of its irritability; had only vomited twice since commenced taking the lime-water in milk.

I ordered the above treatment kept up, and in addition gave the following recipe:

| | | |
|---|---|---|
| ℞ | Acid Salicylic, . . . . . . . | ℈iss. |
| | Spts. Mendereri, . . . . . . . | ℨvij. |
| | Glycerine, . . . . . . . . | ℨi. |
| | Syr. Simp., . . . . . . . . | ℥i. |

M.S.—Teaspoonful every 3 hours.

The next day the father came to town and reported the child's condition considerably improved. It had slept nearly all night, in consequence of which only one dose of the above mixture had been given during the night, the swelling very much reduced and the leg could be rubbed without causing much pain. About three or four hours after this interview Mr. F. summoned me in great haste, saying that he thought the child dying, as it screamed out whenever touched about the chest, and seemed to experience great difficulty and pain in breathing. This news told only too plainly the sad story; metastasis had occurred, and the disease had attacked the muscles of the chest and heart. Before I could reach the patient dissolution had taken place.

This child had never been sick before, and was remarkably stout and large for its age. During the past winter its brother, about five years of age, has had as many as three attacks of rheumatism, one of the attacks quite severe.

The parents of these children, as far as I know, have never had any severe attacks of this disease.

My experience in the use of salicylic acid in rheumatism has been very encouraging; have treated a great many adult cases with invariable success, and one case of a little boy of seven years who recovered entirely within two weeks. I confine my use of the drug solely to the strong and vigorous class of subjects.

UKIAH CITY, CAL., *March* 30, 1880.

